# 424. SARS-CoV-2 Positivity and Genetic Relationships among Cases within Households in the Cascadia Prospective Cohort Study, July 2022 to May 2023

**DOI:** 10.1093/ofid/ofae631.138

**Published:** 2025-01-29

**Authors:** Amanda M Casto, Tara M Babu, Sarah N Cox, Collrane J Frivold, Julia C Bennett, Allison L Naleway, Mark A Schmidt, Jennifer L Kuntz, Cassandra Boisvert, Stephen P Fortmann, Holly C Groom, Richard A Mularski, Sacha L Reich, Neil D Yetz, Leora R Feldstein, Claire Midgley, Melissa Briggs-Hagen, Madison R Hollcroft, Devon McDonald, Mark Drummond, Melissa P MacMillan, Peter D Han, Zack Acker, Sally Grindstaff, Luis Gamboa, Jeremy Stone, Ben Capodanno, Kathryn McCaffrey, Brenna Ehmen, Lani Regelbrugge, Natalie K Lo, Lea Starita, Christina Lockwood, Janet A Englund, Marco Carone, Ana A Weil, Helen Y Chu

**Affiliations:** University of Washington, Seattle, Washington; University of Washington, Seattle, Washington; University of Washington, Seattle, Washington; University of Washington, Seattle, Washington; University of Washington, Seattle, Washington; Kaiser Permanente Center for Health Research, Portland, Oregon; Center for Health Research, Kaiser Permanente Northwest, Portland, Oregon; Kaiser Permanente Center for Health Research, Portland, Oregon; Kaiser Permanente, Portland, Oregon; Kaiser Permanente, Portland, Oregon; Kaiser Permanente Center for Health Research, Portland, Oregon; 1. Kaiser Permanente Center for Health Research, Portland, Oregon, Portland, Oregon; Kaiser Permanente Center for Health Research, Portland, Oregon; Kaiser Permanente Center for Health Research, Portland, Oregon; Centers for Disease Control and Prevention, Atlanta, GA; Centers for Disease Control and Prevention, Atlanta, GA; Centers for Disease Control and Prevention, Atlanta, GA; University of Washington School of Medicine, MOUNTLAKE TERRACE, Washington; University of Washington, Seattle, Washington; University of Washington, Seattle, Washington; University of Washington, Seattle, Washington; University of Washington, Seattle, Washington; Brotman Baty Institute for Precision Medicine, Seattle, Washington; Brotman Baty Institute, Seattle, Washington; Brotman Baty Institute for Precision Medicine, Seattle, Washington; Brotman Baty Institute, University of Washington, Seattle, Washington; Brotman Baty Institute, Seattle, Washington; Brotman Baty Institute, Seattle, Washington; Brotman Baty Institute, Seattle, Washington; Brotman Baty Institute, Seattle, Washington; University of Washington, Seattle, Washington; University of Washington, Seattle, Washington; University of Washington, Seattle, Washington; Seattle Children’s Hospital, Seattle, Washington; University of Washington, Seattle, Washington; University of Washington, Seattle, Washington; University of Washington, Seattle, Washington

## Abstract

**Background:**

Household transmission is a major driver of SARS-CoV-2 spread. Viral genomic sequencing is a powerful tool for evaluation of putative within-household transmission.

**Figure:**
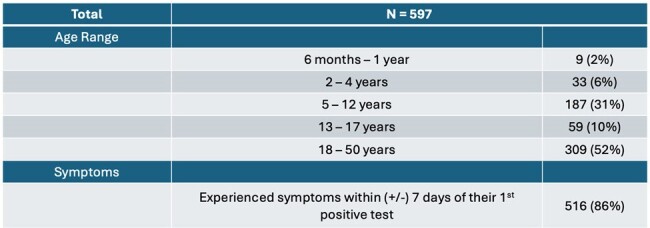

Characteristics of participants from multi-participant households with one or more positive tests for SARS-CoV-2

**Methods:**

CASCADIA, a prospective household cohort study that enrolled individuals in Oregon and Washington state, conducted active surveillance for respiratory viral infection through weekly symptom surveys and self-collected nasal swabs. Additional swabs and surveys were collected when new symptoms were reported or after an initial positive or inconclusive SARS-CoV-2 PCR test. Here we examine all positive SARS-CoV-2 test results from households with > 2 members enrolled in the study. Tests resulting as inconclusive were not considered. We also analyze genomic data generated from these positive swabs, limiting consideration to the first SARS-CoV-2 genome from each person and to households with sequenced samples from > 2 participants collected ≤ 14 days apart. Relationships among samples from the same household were assessed by determining the genetic (hamming) distance between genomes and the Pango lineage of each genome using Nextclade.

**Figure:**
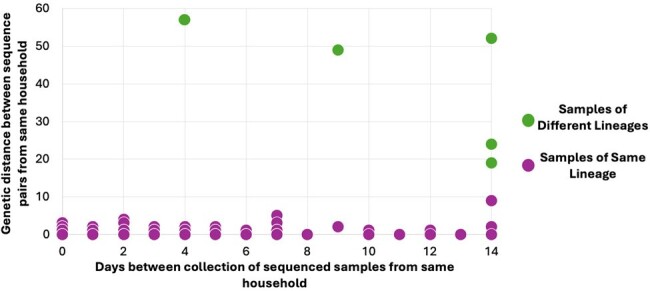

Characteristics of pairs of positive samples from different study participants within the same household. Consideration was limited to the first sequenced sample from each participant and to pairs of samples collected 14 days apart or less.

**Results:**

Between July 2022 to May 2023, 597 participants from 341 households with > 2 participants tested positive for SARS-CoV-2 (Table 1). Positive tests from > 2 participants collected ≤ 14 days apart were observed in 143 of these households. 93 households had sequence data for samples from > 2 persons collected ≤ 14 days apart. 5 (5%) of these households had 2 viral lineages detected with a median of 11 (range 4 – 14) days between detection of the first and second lineages (Figure 1). Two lineages were detected in 3 out of 6 sample pairs collected in the same household exactly 14 days apart. Among the 88 households with one lineage detected, samples from all participants were genetically identical (genetic distance of 0) in 60% (51/88).

**Conclusion:**

SARS-CoV-2 genomes were identical in most households with sequence data from multiple cases detected ≤ 14 days apart. Multiple viral lineages were detected in a subset of the remaining households, suggesting > 1 viral introduction event rather than intra-household transmission. This data will aid in interpretation of household-based studies of SARS-CoV-2 that lack sequence data.

**Disclosures:**

**Mark A. Schmidt, PhD, MPH**, HilleVax: Grant/Research Support|Janssen: Grant/Research Support|Moderna: Grant/Research Support|Pfizer: Grant/Research Support|Vir Biotechnology: Grant/Research Support **Jennifer L. Kuntz, MS, PhD**, Hillevax, Inc: Grant/Research Support **Stephen P. Fortmann, MD**, Pfizer: Grant/Research Support **Holly C. Groom, MPH**, Hillevax: Grant/Research Support|Moderna: Grant/Research Support **Richard A. Mularski, MD, MSHS, MCR**, Pfizer, Inc: Grant/Research Support **Neil D. Yetz, M.P.H.**, N/A: Expert Testimony **Janet A. Englund, MD**, Abbvie: Advisor/Consultant|AstraZeneca: Advisor/Consultant|AstraZeneca: Grant/Research Support|GlaxoSmithKline: Advisor/Consultant|GlaxoSmithKline: Grant/Research Support|Meissa Vaccines: Advisor/Consultant|Merck: Advisor/Consultant|Pfizer: Board Member|Pfizer: Grant/Research Support|Pfizer: Speaker at meeting|SanofiPasteur: Advisor/Consultant|Shinogi: Advisor/Consultant **Helen Y. Chu, MD, MPH**, Abbvie: Advisor/Consultant|Merck: Advisor/Consultant|Vir: Advisor/Consultant

